# Clinical outcomes and hemodynamic performance of Dafodil™ aortic and mitral pericardial bioprosthesis: 1-year results from Dafodil-1 first-in-human trial

**DOI:** 10.1186/s13019-020-01154-7

**Published:** 2020-06-15

**Authors:** C. S. Hiremath, Anil R. Jain, Anurag Garg, Nirmal Gupta, Yugal K. Mishra, Zile Singh Meharwal, Nityanand Thakur, Atul A. Maslekar, Naman Shastri

**Affiliations:** 1 Department of Cardiothoracic Vascular Surgery, Sri Sathya Sai Institute of Higher Medical Sciences, Whitefield, Bengaluru, Karnataka 560066 India; 2 Department of Cardiovascular and Thoracic Surgery, Epic Hospital, Sola, Ahmedabad, Gujarat 380081 India; 3 Department of Cardiovascular and Thoracic Surgery, Dr. D. Y. Patil Medical College, Hospital & Research Centre, Pimpri, Pune, Maharashtra 411018 India; 4 Department of Cardiovascular and Thoracic Surgery, Sanjay Gandhi Postgraduate Institute of Medical Sciences, Raebareli Road, Lucknow, Uttar Pradesh 226014 India; 5 Department of Cardiac Science, Manipal Hospital, Dwarka, New Delhi, 110075 India; 6 Department of Cardiovascular Surgery, Fortis Escorts Heart Institute & Research Centre, Okhla Road, New Delhi, 110025 India; 7 Department of Cardiovascular and Thoracic Surgery, Byramjee Jeejeebhoy Government Medical College and Sassoon General Hospitals, Jay Prakash Narayan road, Pune, Maharashtra 411001 India; 8 Department of Cardiac Surgery – Adult, Narayana Multispeciality Hospital, Rakhial, Ahmedabad, Gujarat 380023 India; 9 Department of Anaesthesia and Intensive Care, Epic Hospital, Sola, Sarkhej - Gandhinagar Highway, Opp. Kargil Petrol Pump, Ahmedabad, Gujarat 380081 India

**Keywords:** Aortic valve, Bioprosthesis, Heart valve prosthesis implantation, Hemodynamic performance, Mitral valve, Quality of life

## Abstract

**Background:**

Bioprosthesis has been increasingly implanted for the treatment of transvalvular disease across the world. A new Dafodil™ pericardial bioprosthesis (Meril Life Sciences Pvt. Ltd., India) recently approved by Conformité Européenne (CE) is a tri-leaflet, stented, bovine valve. The purpose of Dafodil-1 first-in-human trial was to evaluate clinical safety and performance (including hemodynamic parameters) of the Dafodil pericardial bioprosthesis in patients who underwent aortic or mitral valve replacement.

**Methods:**

This prospective, multicenter clinical trial enrolled 60 patients (Aortic: 30 patients; Mitral: 30 patients) from seven sites across India. Safety endpoints were early (≤30 days) and late (> 30 days) mortality and valve-related morbidity. The performance endpoints were hemodynamic performance, improvement in NYHA functional class, and change in the quality of life using SF-12v1 health survey.

**Results:**

From July 2017 to July 2018, 60 patients underwent implantation of the Dafodil pericardial bioprosthesis. Post-operatively, NYHA functional class significantly improved in all the patients (Aortic: 90% NYHA class-I and 10% NYHA class-II; Mitral: 96.55% NYHA class-I and 3.45% NYHA class-II; *P* < 0.001). There was no death in aortic valve replacement patients till 12-month. In mitral valve replacement patients, early mortalities occurred in three patients, and late mortality occurred in one patient; none of these were valve-related. Freedom from all-cause mortality reported was 93.33% at 12-month. Mean aortic pressure gradient decreased from 52.71 ± 24.47 mmHg [with 0.89 ± 0.70 cm^2^ effective orifice area (EOA)] pre-operatively to 14.49 ± 6.58 mmHg (EOA: 1.85 ± 0.27 cm^2^) at 12-month. Overall, the mitral mean pressure gradient and EOA were 4.41 ± 1.69 mmHg and 2.67 ± 0.48 cm^2^, respectively, at 12-month. Significant improvement (*P* < 0.05) in the patients’ quality of life was reported at all follow-ups.

**Conclusions:**

The clinical safety and performance of the Dafodil pericardial bioprosthesis were favourable at 12-month. Moreover, a study with a larger patient population and longer follow-up is warranted to further assess the device.

**Trial registration:**

Dafodil-1 trial has been prospectively registered on 10/07/2017 under Clinical Trial Registry-India (http://www.ctri.nic.in). (Registration number: CTRI/2017/07/009008).

## Background

Prosthetic valve replacement is the only viable option for severe rheumatic and non-rheumatic native valvular heart disease. Valve replacement surgery has been improving the survival and quality of life of the patients for the last six decades [1]. The growing elderly population is associated with an increased incidence of valvular disease. In the Western world, one in every 1000 individuals aged > 65 years undergo valve replacement. According to an estimate, in India, approximately one hundred fifty thousand patients undergo cardiac surgeries, of which 30% are the valve surgeries, including both aortic and mitral valve replacement [2, 3].

Pericardial bioprosthesis have good hemodynamic performance because of their central opening and the flexibility of leaflets. Apart from satisfactory hemodynamic performance, they offer biocompatibility, ease of implantation, and avoidance of long-term anticoagulation [4]. Long-term clinical follow-up also demonstrated excellent performance and improved durability of the bioprosthesis [5–7]. Hence bioprosthesis has been increasingly implanted for the treatment of transvalvular disease.

The Conformité Européenne (CE) approved, Dafodil™ pericardial bioprosthesis (Meril Life Sciences Pvt. Ltd., India) is a tri-leaflet, stented, bovine valve. It is indicated for use in patients whose aortic or mitral valvular disease is sufficiently advanced to warrant a replacement of their natural valve with a prosthetic one. It can also be used in patients with a previously implanted aortic/mitral valve prosthesis, which is no longer functioning adequately and requires replacement. This study was designed to evaluate clinical safety and performance of the Dafodil pericardial bioprosthesis in patients who require replacement of their aortic or mitral valve. Moreover, we also explored the hemodynamic performance of the bioprosthesis in all the patients at 12-month follow-up.

## Methods

Dafodil-1 (CTRI/2017/07/009008) was a first-in-human, prospective, single-arm, and multicentre trial of Dafodil pericardial bioprosthesis which enrolled patients from seven clinical centres across India between July 2017 and July 2018. The study protocol was reviewed and approved by ethics committees of all sites before the initiation of study related activities, and written informed consent was obtained from all the enrolled patients.

A total of 60 patients [aortic valve replacement (AVR): 30 patients & mitral valve replacement (MVR): 30 patients] who required replacement of their aortic or mitral valve with Dafodil pericardial bioprosthesis were enrolled depending upon the inclusion and exclusion criteria. Details on the eligibility criteria are provided in the Appendix.

Surgical technique for implantation of the bioprosthesis, as well as management of antithromboembolic therapy was left to the discretion of the surgeons to reflect the real-world surgical valve replacement as much as possible. Post-operatively, patients were evaluated for the occurrence of events at the following intervals: post-procedure, 1-month, 6-month, 12-month, and annually thereafter up to 5-year. Results of transthoracic echocardiography were evaluated in all the enrolled patients by independent core laboratory (CBCC Global Research LLP, Ahmedabad, India) at pre-procedure, post-procedure, 1-month, 6-month, and 12-month follow-up. Echocardiography parameters were calculated using software DigiView, version 3.7.7.6. Transvalvular pressure gradients were calculated with the modified Bernoulli equation, the aortic effective orifice area (EOA) was derived by using the continuity equation, and the mitral valve area was calculated using the pressure half-time method. Color Doppler imaging (five chamber and four chamber view) was used to assess the presence and degree of aortic and mitral regurgitation. Prosthesis–patient mismatch (PPM), for AVR, was defined as follows: none/mild if EOAi > 0.85 cm^2^/m^2^; moderate if EOAi > 0.65 to ≤0.85 cm^2^/m^2^; and severe if EOAi ≤0.65 cm^2^/m^2^ [8]. PPM for MVR was defined as follows: not clinically significant if EOAi > 1.2 cm^2^/m^2^; moderate if EOAi > 0.9 and ≤ 1.2 cm^2^/m^2^, and as severe if EOAi ≤0.9 cm^2^/m^2^ [9].

The safety endpoints were early (≤30 days) and late (> 30 days) mortality and valve-related morbidity [stroke and transient ischemic attack (TIA), major and minor bleeding, acute kidney injury (AKI), valve thrombosis, structural/non-structural valve deterioration/dysfunction, prosthetic valve endocarditis, conduction disturbances and arrhythmias, mitral valve apparatus damage or dysfunction, explant, hemolysis, study valve-related reoperation]. Early mortality was defined as all-cause mortality at 30 days, depicted by percentages, regardless of the patient’s location, be it home or in a health care facility. Late mortality was defined as all deaths that occurred after 30 postoperative days. The definitions of morbidities are provided in the Appendix. The performance endpoints were hemodynamic performance, improvement in NYHA functional class, and change in the quality of life (QoL) using Short Form (SF)-12v1 Health Survey [10].

### Device description

**Fig. 1:**
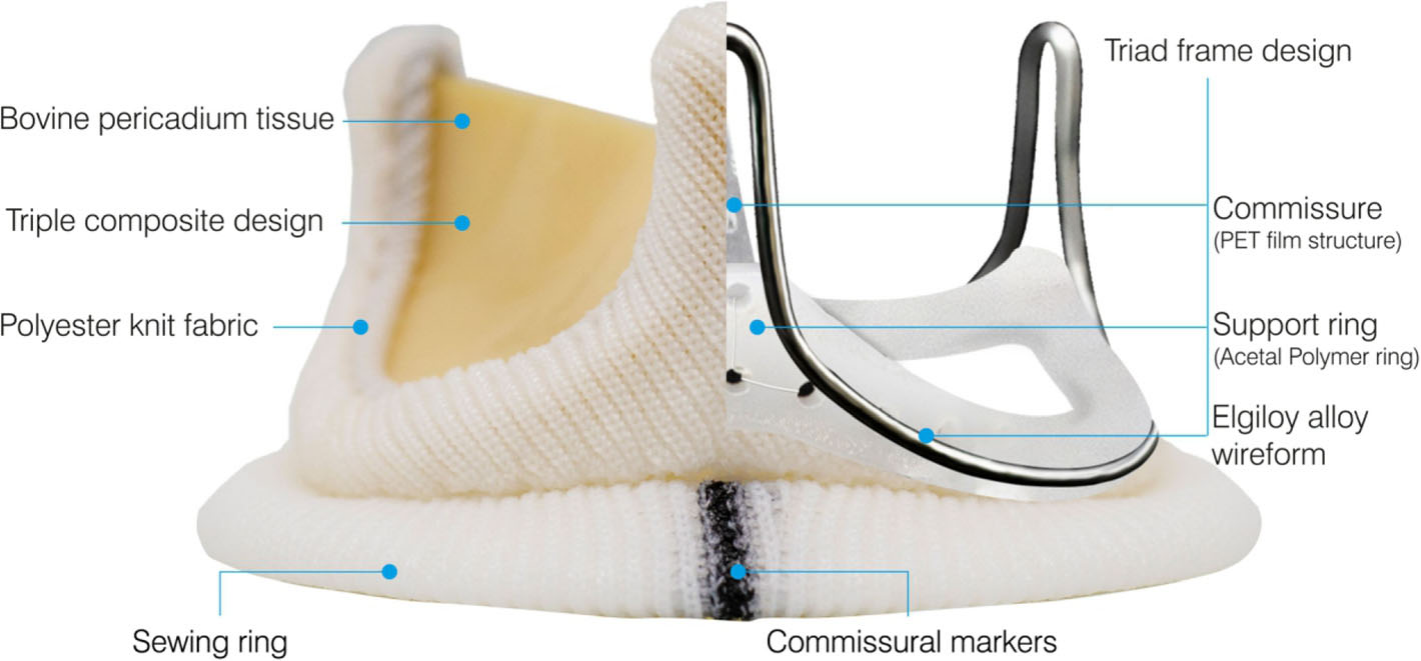
Schematic of Dafodil™ aortic pericardial bioprosthesis

The Dafodil pericardial bioprosthesis is comprised of three leaflets made from bovine pericardial tissue (pre-treated for anti-calcification), a frame, and a sewing ring. The frame is comprised of a polymer ring (support ring), polyethylene terephthalate film structures (posts or commissures), and a frame made of Elgiloy alloy wire-form. These three components of the frame are covered with polyester fabric. The frame is designed to be compliant at the orifice and commissures. A sewing ring made from polyester fabric is attached to the covered frame. There are three contrasting markings on the sewing ring, which aid in proper orientation of the valve. Figure 1 illustrated Dafodil pericardial bioprosthesis (aortic) and its structural components.

‘AntiCa’ is a proprietary anti-calcification process, used during the manufacturing of Dafodil pericardial bioprosthesis to mitigate the risk of calcification. Even after the chemical fixation with glutaraldehyde, the presence of residual calcium in the tissue coupled with a phosphorous from lipids may favour conditions for calcium phosphate crystallization. ‘AntiCa’ process involves exposure of glutaraldehyde fixed bovine pericardial tissue to a proprietary cocktail of a cross-linking agent, denaturant, and surfactant, which targets to chemically extract phospholipids from the cellular components of pericardial tissue and to inactivate the glutaraldehyde resistant organisms. This process reduces bio-burden and renders the tissue resistant to calcification. The durability of the Dafodil pericardial bioprosthesis is supported by the 200 million cycles (equivalent to 5 years in vivo), and biocompatibility of the valve had been evaluated and established by the independent laboratory. The animal study was conducted to evaluate the safety and performance of Dafodil pericardial bioprosthesis in an ovine model. The study results demonstrated hemocompatibility, optimal healing, and long-term durability of the valve till 6 months.

### Statistical analysis

Descriptive statistics were used to summarize the patient population data, operative data, follow-up data, and hemodynamic data. Continuous variables were presented as mean ± standard deviation, and categorical variables were presented as number and percentage. Early adverse event rates (those occurring ≤30 days post-implant) were calculated as the number of early events/total number of subjects, expressed as a percentage. Linearized rates of late adverse events were calculated as the total number of late events (those occurring > 30 days post-implant) divided by the total follow-up time (the sum of accumulated post-operative valve-years), expressed as a percentage. Statistical analysis was performed using the Statistical Package for Social Sciences, version 23 (SPSS, Chicago, IL, USA). Paired data were evaluated using paired t-test for continuous variables. The results were considered significant at *P* < 0.05. Since there is no hypothesis testing in this study; the sample size is not calculated based on the endpoint hypothesis. However, the sample size requirement is determined by assessing the minimal number of patients required to provide reliable and non-trivial results. Hence, the study was designed to enroll 60 patients, including dropout, to evaluate the clinical safety and performance of the Dafodil pericardial bioprosthesis.

## Results

The Dafodil pericardial bioprosthesis was implanted in 60 patients for AVR (*n* = 30 patients) and MVR (*n* = 30 patients). The mean age of the population was 53.95 ± 12.09 years, with a range of 18 to 72 years. Of the patients in AVR group, 17 patients were less than 60 years of age, and one patient was older than 70 years. Among patients implanted with mitral bioprosthesis, 23 patients were less than 60 years of age (including two patients of < 20 years of age), and seven patients were between 61 to 70 years. Concomitant cardiac surgery was performed in 20 patients. Demographic details and baseline clinical characteristics of the study population are outlined in Table 1. The valve sizes used in aortic positions were 19 mm in 11 patients, 21 mm in 13 patients, 23 mm in three patients, and 25 mm in three patients. In MVR group, valve sizes implanted were 25 mm (*n* = 5), 27 mm (*n* = 12), 29 mm (*n* = 3) and 31 mm (*n* = 10).

**Table 1:** Baseline clinical characteristics of the study population

**Demographic characteristics**	**Total** ***n*** = 60 **patients**	**AVR** ***n* = 30 Patients**	**MVR** ***n* = 30 Patients**
Age (years), (mean ± SD)	53.95 ± 12.09	59.83 ± 8.33	48.57 ± 12.63
Age groups, n (%)			
≤ 60 years	40 (66.67)	17 (56.67)	23 (76.67)
61–70 years	19 (31.67)	12 (40.0)	7 (23.33)
> 70 years	1 (1.67)	1 (3.33)	0 (0.00)
Gender, n (%)			
Male	33 (55)	21 (70)	12 (40)
Female	27 (45)	9 (30)	18 (60)
Body mass index (kg/m^2^), (mean ± SD)	23.15 ± 4.83	24.16 ± 4.41	21.58 ± 4.35
STS risk of mortality, % (mean ± SD)	–	1.18 ± 0.63	1.38 ± 0.82
Cardiopulmonary bypass time (minutes), (mean ± SD)	129.56 ± 45.72	148.77 ± 62.81	114.69 ± 27.01
Co-morbidities, n (%)			
Smokers	4 (6.67)	3 (10)	1 (3.33)
Diabetes mellitus	5 (8.33)	4 (13.33)	1 (3.33)
Hypertension	13 (21.67)	9 (30)	4 (13.33)
Previous Cardiac events/surgeries			
Previous MI	1 (1.67)	1 (3.33)	0 (0.00)
Previous PCI	1 (1.67)	1 (3.33)	0 (0.00)
Cerebrovascular events	2 (3.33)	0 (0.00)	2 (6.67)
Previous valvuloplasty	1 (1.67)	0 (0.00)	1 (3.33)
Congestive heart failure	1 (1.67)	1 (3.33)	0 (0.00)
Etiology, n (%)			
Rheumatic	33 (55.00)	11 (36.67)	22 (73.33)
Degenerative	27 (45.00)	19 (63.33)	08 (26.67)
Concomitant procedures, n (%)	20 (100)	10 (50.00)	10 (50.00)
Coronary artery bypass grafts	7 (35.00)	4 (40.00)	3 (30.00)
Mitral valve repair	3 (15)	3 (30.00)	0 (0.00)
Tricuspid valve repair	7 (35.00)	0 (0.00)	7 (70.00)
Aortic root enlargement	2 (10.00)	2 (20.00)	0 (0.00)
Ascending aorta replacement	1 (5.00)	1 (10.00)	0 (0.00)

*AVR* Aortic valve replacement, *MI* Myocardial infarction, *MVR* Mitral valve replacement, *PCI* Percutaneous coronary intervention, *STS* The Society of Thoracic Surgeons

At 12-month, clinical follow-up was completed in 93.33% patients, and total valve years were 56.91 valve years. There was no mortality reported in the AVR group up to 12-month follow-up. In the MVR group, early and late all-cause mortalities reported were three (10%) and one (3.77%/patient-year), respectively; none of them were related to the valve. Out of four mortalities reported at 12-month follow-up, there were two cardiac deaths, which were independent of valve function, whereas another two patients died due to multi-organ failure. Amongst the two cardiac deaths, one was attributed to congestive cardiac failure, and the second death occurred due to biventricular dysfunction followed by cardiopulmonary arrest (patient underwent concomitant tricuspid valve repair). Hence, 12-month all-cause mortality and cardiac mortality free survival among patients was 93.33% (confidence interval 90.0–98.3) and 96.67% (confidence interval 94.1–96.6), respectively.

There were no episodes of stroke/TIA, major/minor bleeding, AKI, valve thrombosis, structural/non-structural valve deterioration/dysfunction, prosthetic valve endocarditis, mitral valve apparatus damage/dysfunction, explant, hemolysis, or study valve-related reoperation up to 12-month. However, one patient in the AVR group required permanent pacemaker implantation.

**Fig. 2:**
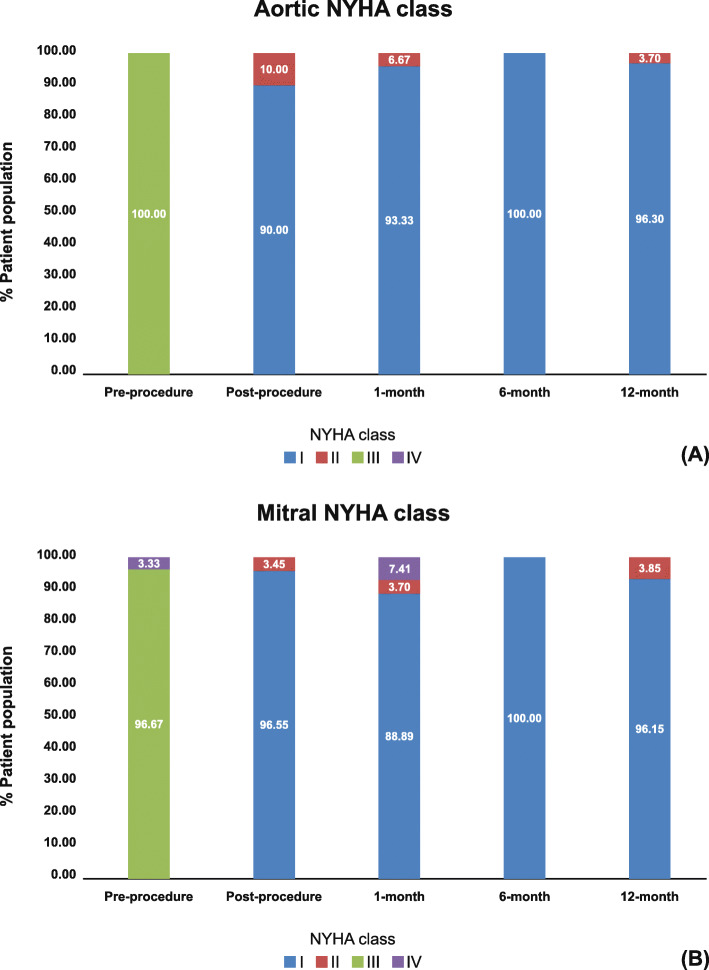
(**a**) Distribution of NYHA functional class among patients implanted with aortic bioprosthesis at pre-procedure, and post-procedure till 12-month; (**b**) Distribution of NYHA functional class among patients implanted with mitral bioprosthesis at pre-procedure, and post-procedure till 12-month

**Fig. 3:**
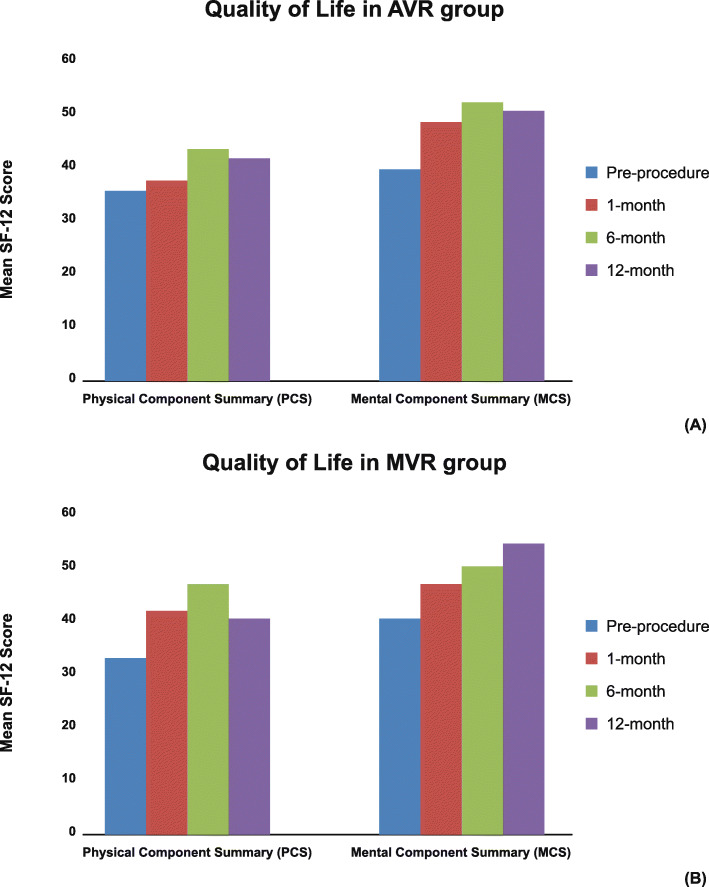
(**a**) Outcomes of PCS and MCS scores of SF12v1 at pre-procedure, and post-procedure till 12-month in AVR group (**b**) Outcomes of PCS and MCS scores of SF12v1 at pre-procedure, and post-procedure till 12-month in MVR group

Pre-operatively, all the patients had NYHA functional class-III in AVR-group (Fig. 2a), whereas 96.67% patients had functional class-III, and 3.33% had functional class-IV in MVR-group (Fig. 2b). Post-operatively, NYHA functional class significantly improved in all the patients (AVR 90% NYHA class-I and 10% NYHA class-II, *P* < 0.001; MVR 96.55% NYHA class-I and 3.45% NYHA class-II, *P* < 0.001) compared to baseline. At 12-month follow-up, NYHA class-I was found in 96.30% and 96.15% patients of AVR and MVR group, respectively. Mean pre-operative SF-12v1 scores showed impaired QoL, both for physical component summary (PCS) score (aortic: 35.98 ± 7.22; mitral: 32.50 ± 6.76) and mental component summary (MCS) score (aortic: 40.43 ± 8.92; mitral: 40.87 ± 8.60), which significantly improved (*P* < 0.05) till 12-month follow-up for both AVR (Fig. 3a) and MVR groups (Fig. 3b).

**Fig. 4:**
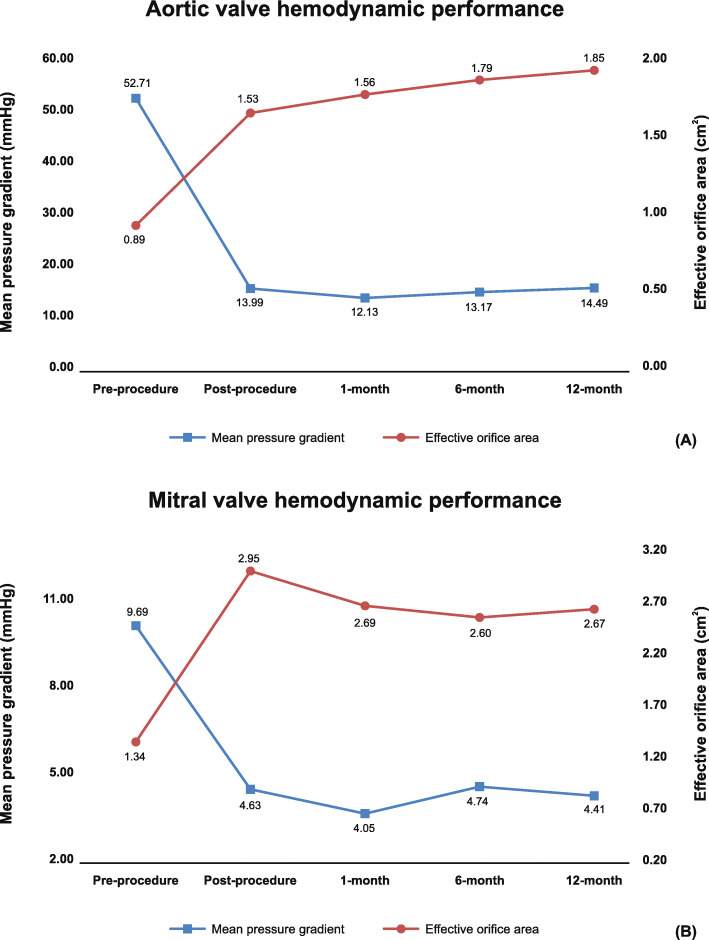
(**a**) Aortic valve hemodynamic performance among patients implanted with aortic bioprosthesis at pre-procedure, and post-procedure till 12-month; (**b**) Mitral valve hemodynamic performance among patients implanted with mitral bioprosthesis at pre-procedure, and post-procedure till 12-month

**Fig. 5:**
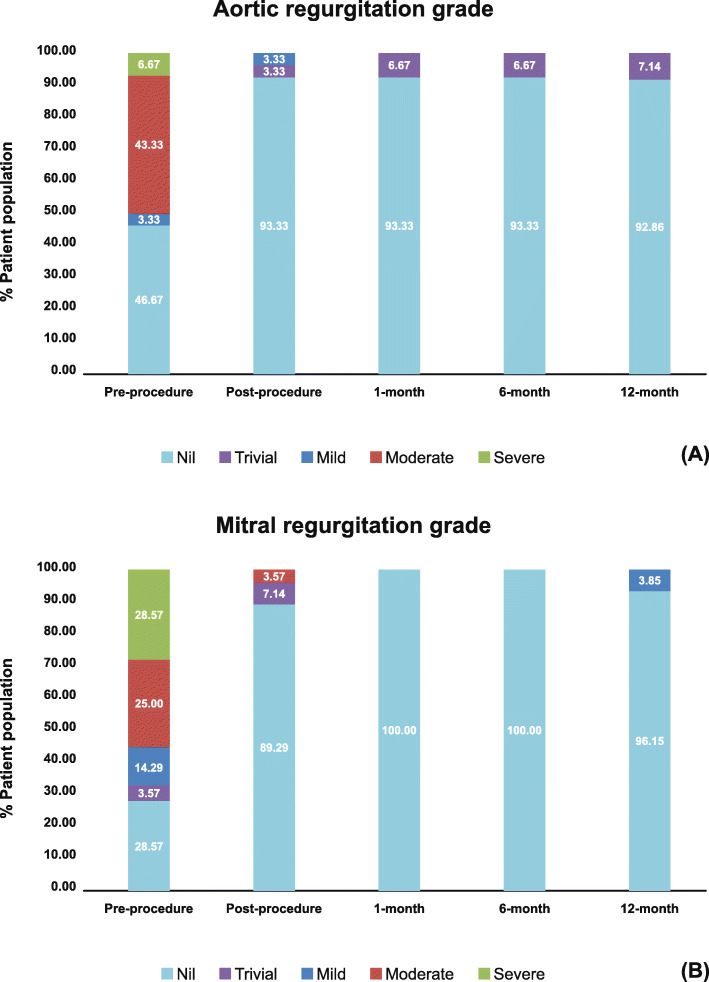
(**a**) Aortic regurgitation among patients implanted with aortic bioprosthesis at pre-procedure, and post-procedure till 12-month; (**b**) Mitral regurgitation among patients implanted with mitral bioprosthesis at pre-procedure, and post-procedure till 12-month

Tables 2 and 3 illustrate the hemodynamic performance of the different valve sizes implanted at aortic and mitral valve position along with cases of PPM till 12-month. There was significant improvement (*P* < 0.001) in mean pressure gradients and EOA of both AVR (Fig. 4a) and MVR (Fig. 4b) groups post-procedure, and over the 12-month compared to pre-procedure. Overall, 90% patients in the AVR group and 92.31% patients in the MVR group are free from PPM at 12-month follow-up. In AVR group, 50% patients had moderate/severe aortic regurgitation pre-operatively (Fig. 5a). However, only 7.14% patients had trivial regurgitation at 12-month follow-up in the AVR group. Pre-operatively, moderate/severe mitral regurgitation was observed in 53.57% patients (Fig. 5b). At 12-month follow-up, there was no regurgitation in 96.15% patients in the MVR group.

**Table 2:** Hemodynamic performance of the study device in patients with aortic valve replacement

**Parameter**	**Valve size (N)**	**Pre-procedure**	**Discharge**	**1-Month**	**6-Month**	**12-Month**
Mean Pressure Gradient (mmHg)	19 (11)	59.94 ± 24.05	16.02 ± 6.47	14.07 ± 3.87	14.70 ± 3.18	16.50 ± 7.52
	21 (13)	58.24 ± 18.63	14.51 ± 5.45	12.14 ± 3.02	13.73 ± 4.28	14.66 ± 5.80
	23 (3)	22.49 ± 14.42	10.13 ± 1.23	9.39 ± 0.61	7.42 ± 2.48	7.49 ± 2.25
	25 (3)	24.23 ± 24.04	9.70 ± 2.30	8.99 ± 2.12	11.41 ± 3.44	13.47 ± 5.57
	Overall (30)	52.71 ± 24.47	13.99 ± 5.60	12.13 ± 3.49	13.17 ± 4.15	14.49 ± 6.58
Peak Pressure Gradient (mmHg)	19 (11)	92.04 ± 33.42	27.19 ± 9.83	24.55 ± 6.12	26.04 ± 5.14	27.71 ± 11.10
	21 (13)	91.49 ± 24.86	24.72 ± 8.06	22.35 ± 5.16	24.05 ± 6.17	24.55 ± 8.94
	23 (3)	39.35 ± 29.20	17.80 ± 1.91	16.66 ± 1.18	14.13 ± 5.13	13.21 ± 2.8
	25 (3)	38.48 ± 36.22	15.54 ± 3.19	15.60 ± 3.46	20.35 ± 8.31	24.07 ± 14.25
	Overall (30)	82.30 ± 34.93	23.75 ± 8.64	22.03 ± 6.02	23.33 ± 6.67	23.25 ± 10.80
Effective Orifice Area (cm^2^)	19 (11)	0.72 ± 0.21	1.59 ± 0.67	1.48 ± 0.31	1.73 ± 0.22	1.78 ± 0.28
	21 (13)	0.68 ± 0.10	1.47 ± 0.55	1.49 ± 0.20	1.75 ± 0.30	1.83 ± 0.23
	23 (3)	1.32 ± NA	1.38 ± 0.11	1.88 ± 0.40	2.08 ± 0.03	2.32 ± 0.13
	25 (3)	2.48 ± 1.97	1.60 ± 0.24	1.92 ± NA	1.83 ± 0.14	1.84 ± 0.11
	Overall (30)	0.89 ± 0.70	1.53 ± 0.54	1.56 ± 0.30	1.79 ± 0.25	1.85 ± 0.27
Effective Orifice Area Index (cm^2^/m^2^)	19 (11)	0.46 ± 0.14	0.98 ± 0.47	0.96 ± 0.20	1.10 ± 0.14	1.16 ± 0.22
	21 (13)	0.40 ± 0.09	0.89 ± 0.37	0.93 ± 0.14	1.05 ± 0.27	1.08 ± 0.22
	23 (3)	0.85 ± NA	0.81 ± 0.18	1.20 ± 0.24	1.25 ± 0.12	1.48 ± 0.06
	25 (3)	1.41 ± 1.08	0.93 ± 0.18	1.15 ± NA	1.06 ± 0.11	1.07 ± 0.10
	Overall (30)	0.54 ± 0.39	0.93 ± 0.38	0.99 ± 0.19	1.09 ± 0.20	1.14 ± 0.22
PPM, n/N (%)	None (EOAi > 0.85 cm^2^/m^2^)	–	20/30 (66.67%)	28/30 (93.33%)	25/30 (83.33%)	27/30 (90%)
	Moderate (EOAi > 0.65 to ≤0.85 cm^2^/m^2^)	–	9/30 (30%)	2/30 (6.67%)	5/30 (16.67%)	3/30 (10%)
	Severe (EOAi ≤0.65 cm^2^/m^2^)	–	1/30 (3.33%)	0/30 (0%)	0/30 (0%)	0/30 (0%)
	Total (EOAi < 0.85 cm^2^/m^2^)	–	10/30 (33.33%)	2/30 (6.67%)	5/30 (16.67%)	3/30 (10%)

*NA* Not applicable, *PPM* Prosthesis–patient mismatch [Pre-operatively, all the patients had aortic regurgitation (alone or in combination with stenosis) in 23 mm and 25 mm groups]

**Table 3:** Hemodynamic performance of the study device in patients with mitral valve replacement

**Parameter**	**Valve size (N)**	**Pre-procedure**	**Discharge**	**1-Month**	**6-Month**	**12-Month**
Mean Pressure Gradient (mmHg)	25 (5)	15.03 ± 4.11	7.64 ± 1.61	5.55 ± 2.16	7.29 ± 2.06	5.63 ± 1.99
	27 (12)	7.40 ± 3.02	4.08 ± 1.63	3.62 ± 0.93	4.56 ± 1.60	4.20 ± 1.88
	29 (3)	12.91 ± 7.20	4.3 ± 0.49	3.45 ± 0.21	3.15 ± 0.94	3.87 ± 1.76
	31 (10)	8.95 ± 3.80	4.08 ± 1.22	4.21 ± 1.22	4.24 ± 1.19	4.06 ± 1.14
	Overall (30)	9.69 ± 4.71	4.63 ± 1.85	4.05 ± 1.27	4.74 ± 1.88	4.41 ± 1.69
Peak Pressure Gradient (mmHg)	25 (5)	26.15 ± 8.80	15.80 ± 3.93	12.1 ± 4.07	16.83 ± 6.02	12.31 ± 5.18
	27 (12)	15.48 ± 5.18	9.43 ± 3.46	8.71 ± 3.00	11.41 ± 1.73	9.22 ± 2.35
	29 (3)	24.84 ± 7.00	10.00 ± 1.56	8.15 ± 1.03	6.81 ± 2.24	7.67 ± 2.19
	31 (10)	16.99 ± 3.91	9.21 ± 3.57	8.44 ± 3.24	8.85 ± 2.22	8.89 ± 3.77
	Overall (30)	18.67 ± 6.86	10.37 ± 3.98	8.95 ± 3.12	10.86 ± 4.26	9.56 ± 3.66
Effective Orifice Area (cm^2^)	25 (5)	0.98 ± 0.13	2.50 ± 0.48	2.52 ± 0.72	2.45 ± 0.54	2.37 ± 0.44
	27 (12)	1.20 ± 0.61	3.10 ± 0.64	2.61 ± 0.35	2.67 ± 0.51	2.68 ± 0.55
	29 (3)	1.93 ± 0.24	3.85 ± 1.38	3.46 ± 1.06	2.84 ± 0.53	2.86 ± 0.43
	31 (10)	1.50 ± 1.13	2.67 ± 0.70	2.57 ± 0.40	2.49 ± 0.27	2.78 ± 0.42
	Overall (30)	1.34 ± 0.77	2.95 ± 0.80	2.69 ± 0.57	2.60 ± 0.43	2.67 ± 0.48
Effective Orifice Area Index (cm^2^/m^2^)	25 (5)	0.58 ± 0.03	1.50 ± 0.20	1.43 ± 0.39	1.38 ± 0.26	1.38 ± 0.24
	27 (12)	0.83 ± 0.33	2.14 ± 0.55	1.85 ± 0.26	1.82 ± 0.40	1.86 ± 0.52
	29 (3)	1.30 ± 0.17	2.52 ± 0.59	2.29 ± 0.48	1.94 ± 0.57	1.88 ± 0.47
	31 (10)	0.88 ± 0.60	1.75 ± 0.63	1.67 ± 0.42	1.58 ± 0.26	1.79 ± 0.36
	Overall (30)	0.86 ± 0.43	1.96 ± 0.60	1.79 ± 0.41	1.69 ± 0.38	1.75 ± 0.44
PPM, n/N (%)	None (EOAi > 1.2 cm^2^/m^2^)	–	28/29 (96.55%)	26/27 (96.29%)	24/26 (92.31%)	24/26 (92.31%)
	Moderate (EOAi > 0.9 and ≤ 1.2 cm^2^/m^2^)	–	0/29 (0%)	1/27 (3.70%)	2/26 (7.69%)	2/26 (7.69%)
	Severe (EOAi ≤0.9 cm^2^/m^2^)	–	1/29 (3.44%)	0/27 (0%)	0/26 (0%)	0/26 (0%)
	Total (EOAi < 1.2 cm^2^/m^2^)		1/29 (3.44%)	1/27 (3.70%)	2/26 (7.69%)	2/26 (7.69%)

*PPM* Prosthesis–patient mismatch

## Discussion

The Dafodil-1 trial establishes safety and performance of the Dafodil pericardial bioprosthesis in patients undergoing valve replacement, as evident by the low rate of all-cause mortality along with significant improvement in NYHA functional class, QoL, and hemodynamic performance. The absence of valve-related mortality seems noteworthy. Moreover, the surgical technique used for the implantation of the valve (for AVR and MVR) as well as post-operative anticoagulation therapy was left to surgeons’ discretion. Hence, these clinical outcomes reflect contemporary surgical outcomes of AVR and MVR.

It should be noted that in the present study, considerably higher proportion of patients were aged less than 60 years. Although mechanical prosthetic valves are preferred for surgical valve replacement in younger patients, it requires repeated hospital visits to assess the estimation of adequate anticoagulation as well as for cinefluoroscopy (to measure mechanical valve function). However, in India, younger patients require surgical valve replacement as a consequence of rheumatic heart disease [11]. Majority of these patients are based in rural areas to whom repeated hospital visits could not be feasible or expensive. Moreover, the life expectancy in India is 61.1 years, and the linearized failure rate for bioprosthesis is 0.6% ± 0.08% per patient-year [12, 13]. Hence, the aforementioned practical issues and statistics seem sufficient to justify the implantation of a bioprosthesis in younger patients.

In the present study, none of the patients having Dafodil pericardial bioprosthesis for AVR died at 12-month follow-up. The 30-day mortality of patients receiving Dafodil pericardial bioprosthesis for AVR is similar to that of reported by the studies of other valves [3, 14–16]. Reported 30-days mortality rate of Trifecta (St. Jude Medical Inc., MN, USA), and Carpentier-Edwards Perimount Magna Ease (Edwards Lifesciences, Irvine, CA) bioprostheses was 0 and 2%, respectively [15]. The 30-day mortality rate was 1% among patients receiving Avalus valve (Medtronic, Minneapolis, MN, USA) [16]. In contrast to the aforementioned studies, none of the patients of the present study experienced all-cause or valve-related mortality, valve thrombosis, thromboembolism, endocarditis, bleeding events, or reoperation at 12-month follow-up. However, interpretation of the results needs careful consideration of the small sample size of the present study.

Extensive comorbidities, previous valve operations, complex jet, and mitral stenosis led to MVR in approximately 30% of the patients with mitral valve disease [17–19]. In the present study, early mortality rate among patients treated for MVR was found to be 10%, which is comparable to the reported early mortality rate of 10% for Mosaic mitral bioprosthesis (Medtronic, Minneapolis, MN, USA) by Celiento et al. [20] Bourguignon et al. reported 3.3% valve-related deaths within 30-days for Carpentier-Edwards Perimount bioprosthesis in the mitral position [21]. Contrary to that, 0% valve-related death reported in the MVR group of the present study. The linearized rates of late deaths were reported 2.48%/patient-year and 5.8%/patient-year for Carpentier-Edwards Perimount in the mitral position [21, 22]. The parallel event rate (3.77%/patient-year) was observed for patients undergoing MVR with Dafodil pericardial bioprosthesis.

At 12-month follow-up, NYHA functional class significantly improved in all the patients, and none of the patients had NYHA functional class-IV or III, which is comparable to the results reported by other studies [14, 16]. Unavailability of the SF-12v1 assessment for the general Indian population or patients of valvular heart diseases renders difficulty in comparison of SF-12v1 data of the study. The baseline PCS score of SF-12v1 reported in the present study for the AVR group is quite better, and MCS of SF-12v1 is lower than the reported values of the surgical AVR group of the PARTNER trial [23]. The difference in the score speaks for the difference in the age groups and risk categories of the patients in both the studies; nevertheless, the pattern of improvement in physical health during 12-month is similar [23]. The 12-month PCS (40.34 ± 8.11) and MCS (51.92 ± 9.63) scores of MVR group are comparable to the scores (PCS:48.0 ± 1.4; MCS: 52.8 ± 1.9) reported by Suri et al. for the patients undergoing mitral valve operations [24].

The importance of valve prosthesis hemodynamic performance has been emphasized in previous studies [25, 26]. It has been emphasized that valve hemodynamics should be assessed at least 6-month postoperatively to limit the bias of hemodynamic instability in the immediate postoperative course. Hence, we evaluated the hemodynamic performance of Dafodil pericardial bioprosthesis in all the patients up to 12-month follow-up. Hemodynamic performance of the Dafodil pericardial bioprosthesis at aortic position favourably correlates with the hemodynamic performance of the other valves having mean pressure gradients ranging from 9.4 ± 4.3 mmHg to 12.9 ± 3.8 mmHg and EOA ranging from 1.4 ± 2.4 cm^2^ to 1.7 ± 0.4 cm^2^ at 12-month follow-up [3, 14–16]. The mean mitral gradients and EOA (mitral valve) reported in the present study are comparable with those achieved by other mitral bioprostheses [27–29].

The rate of severe PPM was minimal (3.33% in AVR group and 3.44% MVR group) in the present study and only observed at the time of discharge. Overall PPM rate of the Dafodil aortic pericardial bioprosthesis (10%) at 12-month are parallel to the PPM rates observed with some of the bovine pericardial valves (9.2% with Edwards Perimount Magna aortic bioprosthesis) while drastically lower than the others (75.5% with Medtronic Avalus valve and 50% with Carpentier-Edwards Perimount Magna Ease) [15, 25, 30]. The observed PPM rate for MVR is 7.69% at 12-month in the present study which is lower than the 48.5% overall PPM rate reported by Akuffu et al. for the patients undergoing MVR with different bioprosthesis (Medtronics Hancock II, Medtronics Mosaic, St. Jude Bicor and Carpentier-Edwards perimount) [31].

A few limitations of the study need to be addressed. The Dafodil-1 trial was a single-arm study without an active comparator group. The present analysis assessed the clinical performance of the device in a small cohort of patients at one-year.

## Conclusions

In conclusion, the clinical outcomes of the Dafodil-1 trial at one-year demonstrated acceptable preliminary safety and performance of the Dafodil pericardial bioprosthesis implanted at aortic and mitral positions. Moreover, the hemodynamic profile of Dafodil pericardial bioprosthesis confirmed favorable performance of the valve at aortic and mitral positions. However, large studies with longer follow-up are needed to confirm these findings along with a focus on late valve-related complications and valve deterioration.

## Data Availability

The datasets used are available from the corresponding author on reasonable request.

## Appendix

### Key eligibility criteria of Dafodil-1 study

**Inclusion criteria:** (1) Patient must be 18 years or older, must provide written informed consent prior to study procedures, and must agree to attend all follow-up assessments for up to 5 years. (2) STS scores < 4% (Low Risk). (3) Patients diagnosed with aortic/mitral disease requiring valve replacement based on pre-operative evaluation (Medical history, PT/INR or PTT, physical examination, echocardiography, and CBC). (4) Patient who had significant aortic/mitral stenosis or aortic/mitral regurgitation or patient is subjected for aortic/mitral valve replacement due to combine aortic/mitral lesion (stenosis & regurgitation). (5) Patients scheduled to undergo planned aortic/mitral valve replacement with or without concomitant bypass surgery or other valvular surgeries.

**Exclusion Criteria:** (1) Patients with active endocarditis/myocarditis or endocarditis/myocarditis within 3 months to the scheduled aortic/mitral replacement surgery. (2) Patients with renal insufficiency as determined by serum creatinine level ≥ 2.5 mg/dL or end-stage renal disease requiring chronic dialysis at the screening visit. (3) Patients with evidence of stroke, cerebrovascular accident, or transient ischemic attack within 6 months prior to planned valve surgery. (4) Patients who had an acute myocardial infarction within 30 days prior to planned valve surgery or left ventricular ejection fraction ≤20% or requirement of concomitant left ventricular assist device placement. (5) Patients with echocardiographic evidence of an intra-cardiac mass, thrombus, or vegetation. (6) Presence of hypertrophic obstructive cardiomyopathy or abnormal calcium metabolism and hyperparathyroidism in patients. (7) Presence of non-cardiac disease limiting life expectancy to less than 5 years or prior/current organ transplant candidate. (8) Patients with leukopenia (WBC < 3.5 * 103/μL), acute anemia (Hgb < 10.0 g/dL or 6 mmol/L) or thrombocytopenia (platelet count < 50 * 103/μL) accompanied by history of bleeding diathesis and coagulopathy. (9) Patients who had hemodynamic or respiratory instability requiring inotropic support, mechanical circulatory support, or mechanical ventilation within 30 days prior to planned valve surgery. (10) Patients who had participated in a study of any investigational drug or device or documented history of substance (drug or alcohol) abuse within the last 5 years prior to the screening date. (11) The patient with known hypersensitivity or contraindication to antiplatelet drugs, anticoagulant drugs, polyethylene terephthalate, elgiloy, and polyester. (12) Patients who were unable to give voluntary informed consent or female subject of childbearing potential who are pregnant, lactating or planning to become pregnant during the duration of participation in study.

### Definitions of morbidities

**Stroke:** Duration of a focal or global neurological deficit ≥24 h; or < 24 h if available neuroimaging documents a new haemorrhage or infarct; or the neurological deficit results in death; **TIA:** Duration of a focal or global neurological deficit < 24 h, any variable neuroimaging does not demonstrate a new haemorrhage or infarct; **Major bleeding:** Overt bleeding either associated with a drop in the haemoglobin level of at least 3.0 g/dL or requiring transfusion of 2 or 3 units of whole blood/RBC, or causing hospitalization or permanent injury, or requiring surgery and does not meet criteria of life-threatening or disabling bleeding; **Minor bleeding:** Any bleeding worthy of clinical mention (e.g. access site haematoma) that does not qualify as life-threatening*,* disabling, or major; **AKI:** Stage 1: Increase in serum creatinine to 150–199% (1.5–1.99 × increase compared with baseline) or increase of > 0.3 mg/dl (> 26.4 mmol/l) or urine output < 0.5 ml/kg/h for > 6 but < 12 h, Stage 2: Increase in serum creatinine to 200–299% (2.0–2.99 × increase compared with baseline) or urine output < 0.5 ml/kg/h for > 12 but < 24 h, Stage 3: Increase in serum creatinine to > 300% (> 3 × increase compared with baseline) or serum creatinine of > 4.0 mg/dl (> 354 mmol/l) with an acute increase of at least 0.5 mg/dl (44 mmol/l) or urine output < 0.3 ml/kg/h for > 24 h or anuria for > 12 h; **Valve thrombosis:** Any thrombus attached to or near an implanted valve that occludes part of the blood flow path, interferes with valve function, or is sufficiently large to warrant treatment; **Structural valve deterioration**: Change in the function of a heart valve substitute resulting from an intrinsic abnormality that causes stenosis or regurgitation (which includes intrinsic changes such as wear, fatigue failure, stress fracture, occluder escape, suture line disruption of components); **Nonstructural valve dysfunction:** Abnormality extrinsic to the heart valve substitute that results in stenosis, regurgitation, and/or heamolytic anemia; **Prosthetic valve endocarditis:** Any infection involving a valve in which an operation has been performed, based on reoperation and autopsy or the Duke endocarditis criteria for Endocarditis (major criteria: 1.Blood culture positive for Infective Endocarditis 2.Oscillating intracardiac mass on valve or supporting structures, in the path of regurgitant jets); **Conduction disturbances and arrhythmias:** Baseline conduction abnormalities, paroxysmal or permanent atrial fibrillation (or flutter), and the presence of permanent pacemaker (e.g. defibrillator, single vs. dual chamber, biventricular); **Mitral valve apparatus damage or dysfunction:** Echocardiographic evidence of new damage (chordae papillary muscle, or to the leaflet) to the mitral valve apparatus or dysfunction; **Explant:** Removal of the study device that had been implanted; **Hemolysis:** The diagnosis of hemolytic anemia is usually established if three major criteria are present: (a) unexplained anemia, and (b) signs of accelerated RBCs production in the bone marrow (eg, high reticulocyte count), and (c) signs of RBCs destruction; **Study Valve related reoperation:** Reintervention is any surgical or percutaneous interventional catheter procedure that repairs, otherwise alters or adjusts, or replaces a previously implanted prosthesis or repaired valve.

## Abbreviations

AKI: Acute Kidney Injury
AVR: Aortic Valve Replacement
CBC: Complete Blood Count
CTRI: Clinical Trial Registration Number
EOA: Effective Orifice Area
MCS: Mental Component Summary
MI: Myocardial Infarction
MVR: Mitral Valve Replacement
PCI: Percutaneous Coronary Intervention
PCS: Physical Component Summary
PPM: Prosthesis–patient mismatch
PT/IN: Prothrombin Time/ International Normalized Ratio
PTT: Partial Thromboplastin Time
QoL: Quality of Life
RBC: Red Blood Cells
SF-12v1: Short Form-12 version 1
SPSS: Statistical Package for Social Sciences
STS: The Society of Thoracic Surgeons
TIA: Transient Ischemic Attack
